# Efficacy and Safety of a Novel Oil Pulling Oral Rinse for Teeth Whitening: A Double-Blind, Randomised, Placebo-Controlled Trial

**DOI:** 10.7759/cureus.118502

**Published:** 2026-10-24

**Authors:** Chetan Patel, Ashwin Prabhu, Deepa Subramanian

**Affiliations:** 1 Pharmaceutical Sciences, University of Greenwich, Chatham Maritime, GBR; 2 Orthodontics, Rajalakshmi Hospital and Research Center, Bangalore, IND; 3 Clinical Research, Syncretic Clinical Research Services Private Limited, Bangalore, IND

**Keywords:** coconut oil, cosmetic dentistry, essential oils, medium-chain triglyceride (mct) oil, natural tooth whitening, oil pulling, oral rinse, randomized controlled trial, vita shade

## Abstract

Aim

Conventional peroxide-based tooth-whitening systems are effective but are frequently associated with tooth sensitivity and gingival irritation, prompting interest in potentially gentler alternatives. Medium-chain triglyceride (MCT) oil combined with essential oils has been proposed as a possible approach to reducing extrinsic tooth discolouration, but controlled clinical evidence remains limited. This study aimed to evaluate the whitening efficacy and safety of a novel MCT-based oil pulling oral rinse containing an essential oil blend compared with a placebo in adults with tooth shade A2 or darker.

Materials and methods

In this double-blind, randomised, placebo-controlled, single-centre trial, 100 adults (18-65 years) with a baseline tooth shade of A2 or darker were randomised 1:1 to receive the active oil pulling rinse (n=50) or a matched placebo rinse (n=50). Participants self-administered 10 mL of the assigned rinse each morning before toothbrushing for 4 weeks. Tooth shade was assessed at screening, Week 2, and Week 4 using a VITA classical shade guide (VITA Zahnfabrik, Bad Säckingen, Germany), converted to a 16-point Munsell-based numeric scale, and compared between groups using the Wilcoxon test. Safety was assessed through physical and oral examination, vital signs, haematology and biochemistry, and adverse event monitoring.

Results

All 100 randomised participants completed the study with no dropouts or protocol deviations. Mean (SD) tooth-whiteness scores in the active group increased from 5.99 (2.627) at screening to 6.97 (2.769) at Week 2 and 7.29 (2.832) at Week 4, whereas placebo scores remained essentially unchanged (6.16 (2.419) at screening, 6.16 (2.421) at Week 2, - 6.22 (2.462)) at Week 4. Between-group differences favoured the active rinse at Week 2 and Week 4 (both p<0.0001), with continued significant improvement between Week 2 and Week 4 (p=0.0008). Treatment compliance was 100% in both arms; 92% of active-group and 90% of placebo-group participants rated tolerability as excellent. No adverse events and no clinically significant changes in physical examination, oral examination, vital signs, or laboratory parameters were observed in either group. Satisfaction with treatment was markedly higher in the active group.

Conclusion

The active oil pulling oral rinse containing MCT oil and an essential oil blend produced statistically significant, progressive improvement in tooth whiteness over four weeks compared with placebo, with an excellent safety and tolerability profile. These findings support the investigated oil pulling rinse as a viable, gentle adjunct for cosmetic tooth whitening, although confirmation in larger, multi-centre studies is warranted.

## Introduction

A whiter smile is among the most frequently requested cosmetic dental outcomes, and public demand for tooth-whitening products and procedures continues to grow [1-3]. Tooth discolouration is broadly classified as intrinsic or extrinsic. Intrinsic discolouration may arise from factors such as tetracycline exposure, ageing, excessive fluoride exposure, or genetics, whereas extrinsic discolouration may result from smoking, chewing tobacco, dietary pigments, certain metals, and some medications. Teeth whitening can be achieved either by physically removing surface stains or by chemically degrading chromogens on and within the tooth structure [2-3]. Since the introduction of home- and office-based bleaching systems in the late twentieth century, hydrogen peroxide and carbamide peroxide have remained the principal bleaching agents in both take-home over-the-counter (OTC) products, toothpastes, strips, gels, and mouth rinses, and professional in-office treatments [4-5]. Carbamide peroxide dissociates into urea and hydrogen peroxide; the hydrogen peroxide penetrates enamel and dentine and generates reactive oxygen species that oxidise chromophores, reducing their colour intensity [6-8].

Although effective, peroxide-based whitening is commonly associated with adverse effects and discomfort, the occurrence and severity of which are influenced by peroxide concentration, exposure time, individual susceptibility, and treatment protocol [9-10]. Its oxidative mechanism may induce the release of inflammatory mediators, commonly manifesting as tooth sensitivity, while direct contact may cause transient gingival irritation, blanching, and soft-tissue discomfort. Prolonged exposure to high concentrations of peroxide has also been associated with enamel demineralisation and ultrastructural changes, although the clinical significance and persistence of these effects remain uncertain [11-16]. These tolerability concerns have driven interest in potentially milder, non-peroxide and natural alternatives.

Oil pulling, the traditional practice of swishing oil in the mouth, has re-emerged as a potential tooth whitening approach, particularly when formulated with medium-chain triglyceride (MCT) oil and a selected blend of essential oils [17]. Several mechanisms have been proposed: The mechanical action of swishing may dislodge debris and surface stain without relying on abrasion [17]; the lipophilic character of MCT oil may help suspend hydrophobic plaque and biofilm components associated with discolouration [18]; and the antimicrobial properties of the essential oil blend may reduce chromogenic bacteria, while its potential anti-inflammatory effects may support gingival health [19]. Despite this plausible rationale, controlled clinical evidence for the tooth-whitening effects of oil pulling rinses remains sparse.

We therefore conducted a double-blind, randomised, placebo-controlled trial to evaluate the whitening efficacy and safety of a coconut-mint oil pulling oral rinse containing MCT oil and an essential oil blend in comparison with a matched placebo in adults with tooth shade A2 or darker. The primary objective was to evaluate tooth-whitening efficacy by measuring changes in tooth-whiteness scores using the VITA classical shade guide (VITA Zahnfabrik, Bad Säckingen, Germany), with shade tabs converted to a 16-point numerical scale, at baseline, Week 2, and Week 4. Changes in scores were evaluated from baseline to Week 2, baseline to Week 4, and Week 2 to Week 4. The secondary objectives were to assess treatment compliance, overall tolerability, patient-reported treatment satisfaction, and safety through physical and oral examinations, vital signs, laboratory assessments, and adverse-event monitoring.

## Materials and methods

Trial design and oversight

This was a double-blind, randomised, placebo-controlled, single-centre, two-arm, superiority trial conducted at Rajalakshmi Hospital, Bangalore, Karnataka, India, between June and July 2025 (first participant enrolled: 12 June 2025; last participant’s last visit: 16 July 2025). The trial was conducted by Syncretic Clinical Research Services Private Limited (Bangalore, India) in accordance with Good Clinical Practice (GCP) and the ethical principles of the Declaration of Helsinki. The protocol and associated documents (Investigator’s brochure, informed consent forms and translations, case report forms, investigator undertakings, and insurance certificate) were approved by the Rajalakshmi Hospital Institutional Ethics Committee (Approval number: RH/IEC/AP/11/23/05/2025) on 23 May 2025. The trial was prospectively registered with the Clinical Trials Registry-India (CTRI/2025/06/088449). The study was reported in accordance with the CONSORT 2010 statement (Figure 1) [20]. Written informed consent was obtained from all participants before any study procedures were performed.

**Figure 1:**
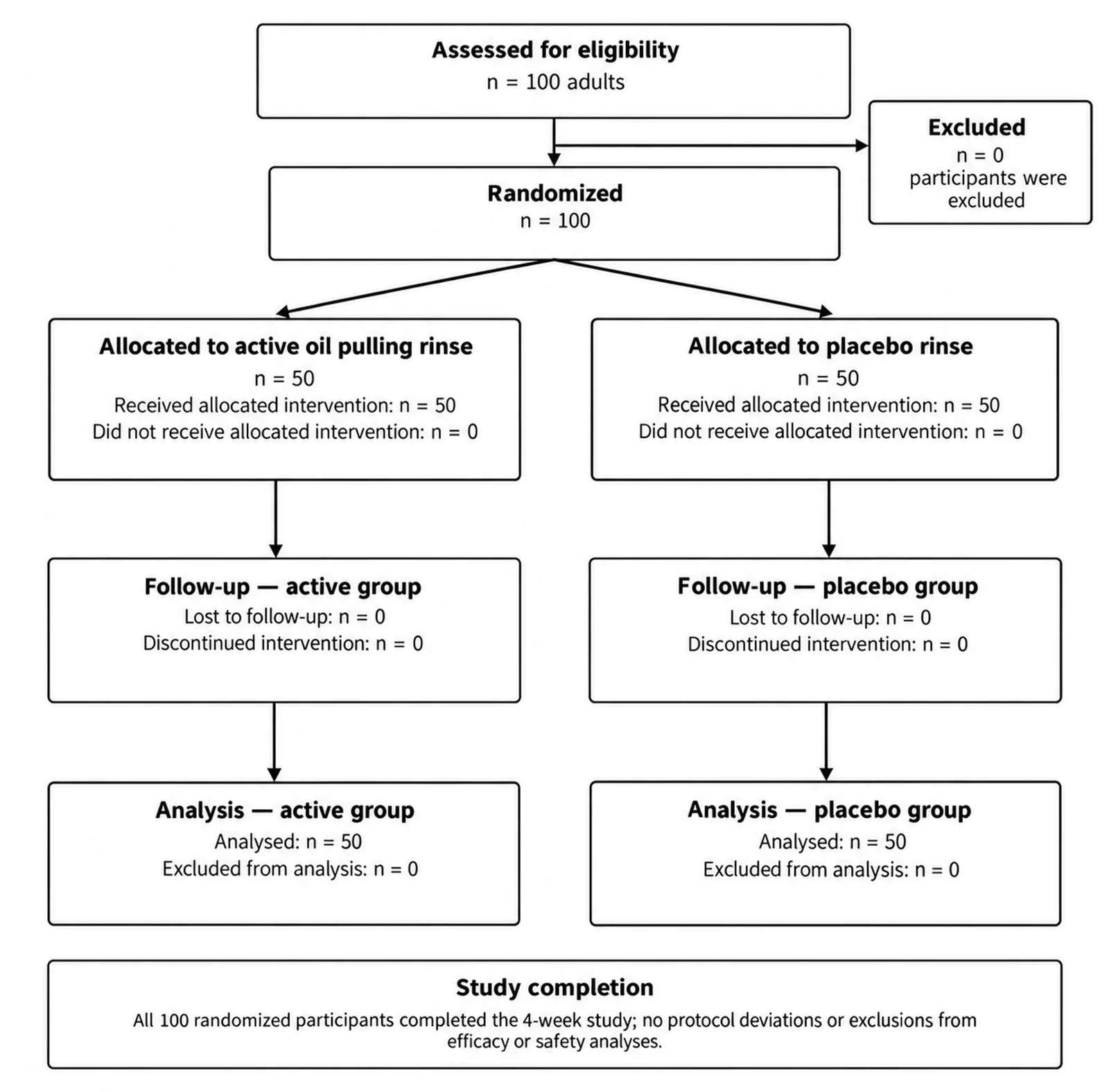
CONSORT diagram CONSORT: Consolidated Standards of Reporting Trials

Participants

Eligible participants were men and women aged 18-65 years who had at least 20 permanent teeth (excluding third molars), were nonsmokers, and were in good general and oral health. All six maxillary anterior teeth were required to be free of caries, restorations, and cervical lesions, and to have a baseline tooth shade of A2 or darker. Participants were also required to have no prosthodontic treatment or periodontal disease. Participants were required to abstain from mouthwash use for one week before screening and throughout the study and to use a single, consistent toothpaste for the study duration.

Key exclusion criteria included intrinsically discoloured teeth (e.g., tetracycline staining or fluorosis); recent use of nonsteroidal anti-inflammatory drugs (NSAIDs) or corticosteroids; significant concomitant disease (malignancy, renal, hepatic, neurological, psychiatric, haematological or gastrointestinal disease, diabetes); pregnancy or breastfeeding; dental procedures or whitening treatments during four weeks before screening; concurrent use of other tooth whitening products or treatments; restorations on anterior teeth; and orthodontic appliances (other than removable retainers). Additionally, individuals who were unable to avoid stain-causing substances, including red wine, coffee, and tea, during the study period were excluded.

Randomisation and blinding

Eligible participants were assigned sequentially to treatment using a computer-generated, simple randomisation schedule prepared centrally. Active and placebo products were packaged in visually identical plastic bottles distinguishable only by a centrally assigned randomisation code; neither participants, the investigator, nor site staff were aware of treatment allocation, maintaining the double-blind design for the duration of the study.

Interventions

Participants randomised to the active arm received a coco-mint oil pulling oral rinse (Gurunanda, USA) containing caprylic/capric/lauric triglycerides (MCT oil) as a base, combined with peppermint, spearmint, clove, cardamom, tea tree, oregano, and fennel oils, stevia leaf extract, and vitamins E, D3, and K2 (all at proprietary concentrations). The placebo rinse was formulated to resemble the active rinse in appearance and viscosity but omitted the MCT oil and essential oil blend that together constituted the active formulation. It comprised water, glycerine, propanediol, xylitol, PEG hydrogenated castor oil, sodium benzoate, flavour, citric acid, and sucralose. Both products were administered identically: 10 mL swished orally for 2 minutes once daily each morning before tooth-brushing for 4 consecutive weeks. Bleaching, professional whitening, antibiotic treatment, and breath-freshening products were prohibited throughout the study; other concomitant medications not known to interact with the study product were permitted at the investigator’s discretion.

Study procedures

Following informed consent, participants underwent screening assessments comprising the collection of demographic data, medical and treatment histories; physical and oral examinations, vital signs measurements, haematological and biochemical testing, urine pregnancy testing (in women of childbearing potential), and baseline tooth-shade evaluation using the VITA classical shade guide (VITA Zahnfabrik, Bad Säckingen, Germany) under standardised outdoor lighting conditions. To minimise environmental and chromatic influences during visual shade matching, participants were seated upright with the maxillary dental arch positioned parallel to the floor. Lipstick and perioral cosmetics were removed, and a neutral blue bib was placed over the participants’ clothing. Shade assessments were conducted under indirect natural daylight, avoiding direct solar glare and artificial operatory lighting, during consistent daytime clinic hours. The examiner was blinded to treatment allocation. Nevertheless, ambient natural daylight may vary between assessment visits, and some residual environmental variability cannot be excluded.

Eligible participants were randomised and enrolled on Day 0 (Visit 2/baseline) and were dispensed the study product with instructions to swish 10 mL of the assigned rinse for 2 minutes once daily in the morning before toothbrushing throughout the 4-week study period. Follow-up visits occurred at Week 2 (Visit 3) and Week 4/end of study (Visit 4), each within a permitted window of ±2 days. At every visit, tooth-shade assessment, oral & physical examination, measurement of vital signs, and reviews of adverse events and concomitant medications were performed; weight and BMI (body mass index) were recorded at Visits 3 and 4. Haematological and biochemical testing and urine pregnancy testing were repeated at Visit 4; overall satisfaction and tolerability questionnaires were also completed, and unused study product was collected for compliance verification.

Outcome measures

The primary efficacy endpoint was change in tooth whiteness from screening to Week 2 and Week 4, and from Week 2 to Week 4. The shade of the upper right central incisor and upper left lateral incisor was matched to the closest VITA tab, and each tab was converted to a numeric value from 1 (C4, darkest) to 16 (B1, lightest), following the conventional value-based shade order in reverse numerical direction (C4=1, A4=2, C3=3, B4=4, A3.5=5, B3=6, D3=7, A3=8, D4=9, C2=10, C1=11, A2=12, D2=13, B2=14, A1=15, and B1=16). Thus, higher scores indicated lighter tooth shades, and the eligibility criterion of A2 or darker corresponded to a score of ≤12. Individual subject scores were calculated by averaging the scores of the assessed teeth. Grossly carious, crowned, or extensively restored teeth were excluded from scoring. A greater positive change in score from baseline indicated greater whitening improvement. Secondary endpoints comprised treatment compliance, overall tolerability, and a six-item overall treatment satisfaction questionnaire (with response options of Very Happy, Happy, NIL, or Not Happy) administered at the end of the study. Safety was assessed throughout via physical examination (general appearance, HEENT (head, eyes, ears, nose, and throat), abdomen, dermatology, respiratory, cardiovascular, gastrointestinal, musculoskeletal, and neurological systems), oral mucosal examination (teeth, gums, tongue, and palate), vital signs (temperature, heart rate, respiratory rate, and systolic and diastolic blood pressure), laboratory parameters (complete blood count with differential, serum glutamic-oxaloacetic transaminase (SGOT), serum glutamate pyruvate transaminase (SGPT), and serum creatinine), and adverse event monitoring at every visit.

Statistical analysis

The study was designed as a superiority trial to evaluate whether the investigational oil pulling oral rinse produced greater improvement in tooth whiteness than placebo over the 4-week treatment period. The sample size was calculated a priori based on the primary efficacy endpoint of improvement in tooth whiteness from baseline to the end of the study. The calculation assumed whitening response proportions of 36% in the active group and 10% in the placebo group, based on previous studies, with 85% statistical power and a two-sided significance level of 0.05. This calculation yielded a requirement of 48 evaluable participants per group. The target sample size was increased to 50 participants per group (100 participants in total) to accommodate anticipated withdrawals.

The assumed 26-percentage-point difference in response proportions was used for sample-size estimation; it should not be interpreted as a separately prespecified clinical superiority margin unless this was explicitly defined in the original protocol. The primary efficacy endpoint was an ordinal tooth-whiteness score. Between-group changes in tooth-whiteness scores were compared using the non-parametric Wilcoxon test. Descriptive statistics, including means, standard deviations, medians, and ranges, were generated for continuous or ordinal outcomes, while categorical variables were summarised using frequencies and percentages. A two-sided p-value of less than 0.05 was considered statistically significant.

The modified intention-to-treat (mITT) population was defined as randomised participants who received the study product and had evaluable post-randomisation data, consistent with the study protocol. The per-protocol (PP) population comprised participants who completed the study without major protocol deviations that could affect the assessment of efficacy. All 100 randomised participants received the assigned study product, completed the 4-week study, and were included in the efficacy and safety analyses. No withdrawals, protocol deviations, or exclusions from the analyses were reported. Consequently, the mITT and PP populations were identical in this study.

An additional retrospective analysis of repeated assessments of the 100 teeth evaluated in the placebo group across Visits 1, 3, and 4 was conducted to assess temporal consistency in visual shade scoring. Exact shade-tab agreement was 92.0% between Visits 1 and 3 and 90.0% between Visits 1 and 4. The corresponding linear-weighted Cohen’s kappa coefficients were 0.980 and 0.976, respectively. The two-way mixed-effects intraclass correlation coefficient for absolute agreement across the three visits was 0.995. These analyses were not prespecified and were intended to assess the consistency of repeated shade assessments; they do not independently establish formal examiner calibration or isolate measurement reliability from the temporal stability of tooth shade.

## Results

Participant disposition and baseline characteristics

A total of 100 adults were screened and enrolled at a single site; all 100 were randomised (50 to the active group and 50 to the placebo group) and completed the 4-week study, with no dropouts, no protocol deviations, or exclusions from the efficacy or safety analyses. Baseline demographic and clinical characteristics were broadly comparable between groups (Table 1), although the placebo group had a somewhat higher proportion of male participants (36 (72.0%) vs 30 (60.0%)). No participant in either group reported a relevant medical history, dental treatment history, or concomitant disease at baseline. Body weight and BMI remained stable and were closely matched between groups at screening.

**Table 1:** Baseline demographic and clinical characteristics

Parameter	Active group (n=50)	Placebo group (n=50)
Age, years, mean (SD)	35.3 (7.43)	34.5 (7.39)
Age, years, median (min–max)	35.5 (22–52)	33.5 (22–54)
Sex, female, n (%)	20 (40.0)	14 (28.0)
Sex, male, n (%)	30 (60.0)	36 (72.0)
Height, cm, mean (SD)	168.0 (5.36)	167.6 (5.36)
Prior medical history, n (%)	0 (0.0)	0 (0.0)
Prior dental treatment history, n (%)	0 (0.0)	0 (0.0)
Concomitant disease, n (%)	0 (0.0)	0 (0.0)

Primary efficacy endpoint: tooth whiteness

Mean tooth-whiteness scores were comparable between groups at screening (active: 5.99 (SD 2.627) vs placebo: 6.16 (SD 2.419)). Over the 4-week treatment period, scores in the active group rose progressively to 6.97 (SD 2.769) at Week 2 and 7.29 (SD 2.832) at Week 4, whereas placebo scores remained unchanged (6.16 (SD 2.421) at Week 2; 6.22 (SD 2.462) at Week 4) (Table 2, Figure 2).

**Table 2:** Descriptive statistics for tooth-whiteness score by visit and treatment group

Visit	Active, mean (SD)	Active, median (min–max)	Placebo, mean (SD)	Placebo, median (min–max)
Screening (baseline)	5.99 (2.627)	6.00 (1.0–11.5)	6.16 (2.419)	6.50 (1.0–11.0)
Week 2 (Visit 3)	6.97 (2.769)	7.25 (2.0–13.0)	6.16 (2.421)	6.50 (1.0–11.0)
Week 4 (Visit 4, EOS)	7.29 (2.832)	7.50 (2.5–14.0)	6.22 (2.462)	6.50 (1.0–11.0)

**Figure 2:**
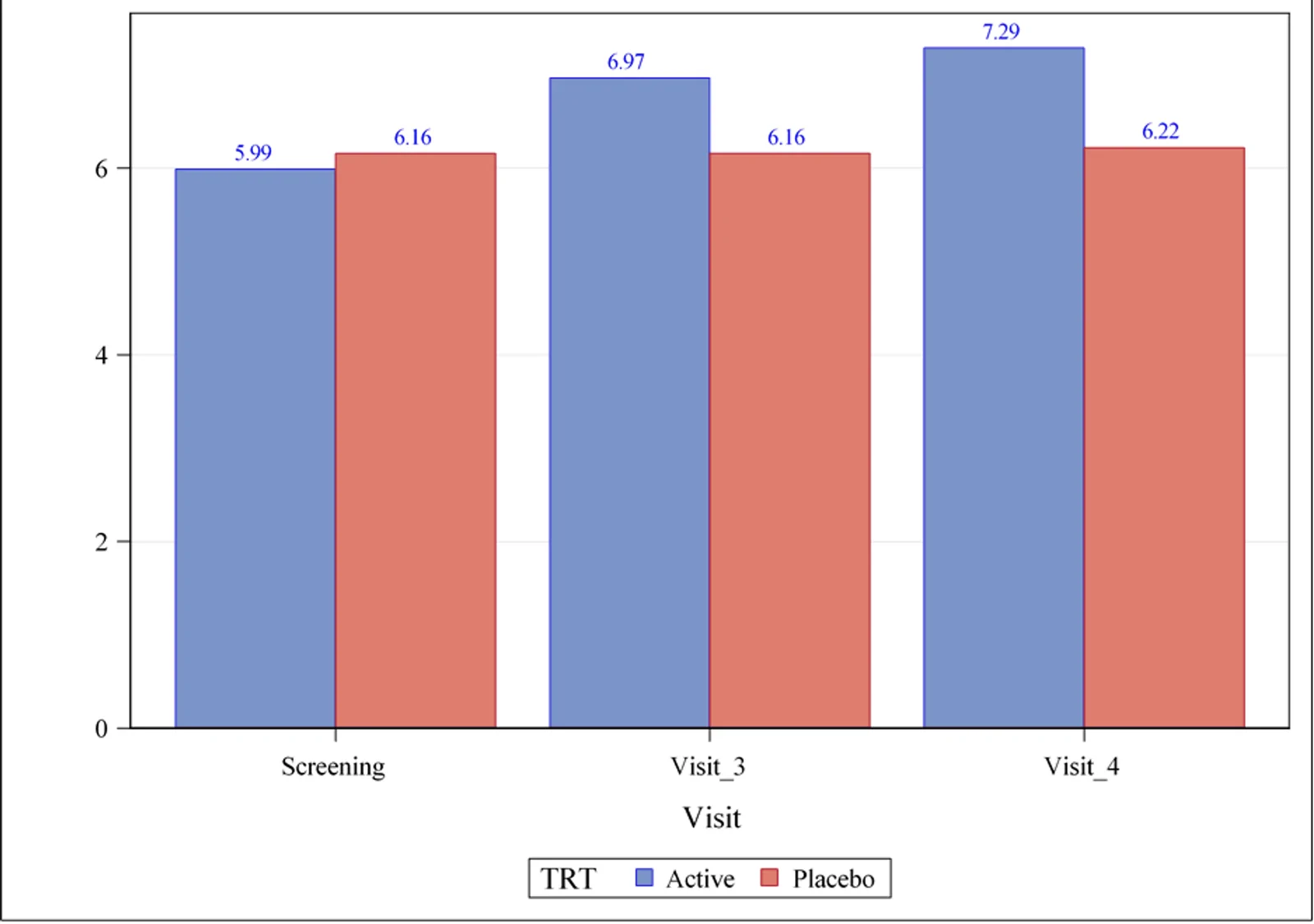
Mean tooth-whiteness scores at screening, Week 2, and Week 4 by treatment group

Between-group changes in tooth-whiteness scores were compared using the non-parametric Wilcoxon test. The improvement in the active group was significantly greater than that in the placebo group from screening to Week 2 (Wilcoxon test statistic = 3599.500; standardised z = 7.9286; p<0.0001) and from screening to Week 4 (Wilcoxon test statistic = 3567.000; standardised z = 7.5973; p<0.0001). A further significant between-group difference in change from Week 2 to Week 4 was observed (Wilcoxon test statistic = 2946.000; standardised z = 3.3409; p=0.0008), indicating continued whitening with ongoing use (Table 3).

**Table 3:** Between-group statistical comparison of tooth-whiteness change (Wilcoxon test)

Comparison	Wilcoxon test statistic	Standardised z value	P value
Change, screening to Week 2 (active vs placebo)	3599.500	7.9286	<0.0001
Change, screening to Week 4 (active vs placebo)	3567.000	7.5973	<0.0001
Change, Week 2 to Week 4 (active vs placebo)	2946.000	3.3409	0.0008

The changes in mean tooth-whiteness scores over the four-week study period are illustrated in Figure 3.

**Figure 3:**
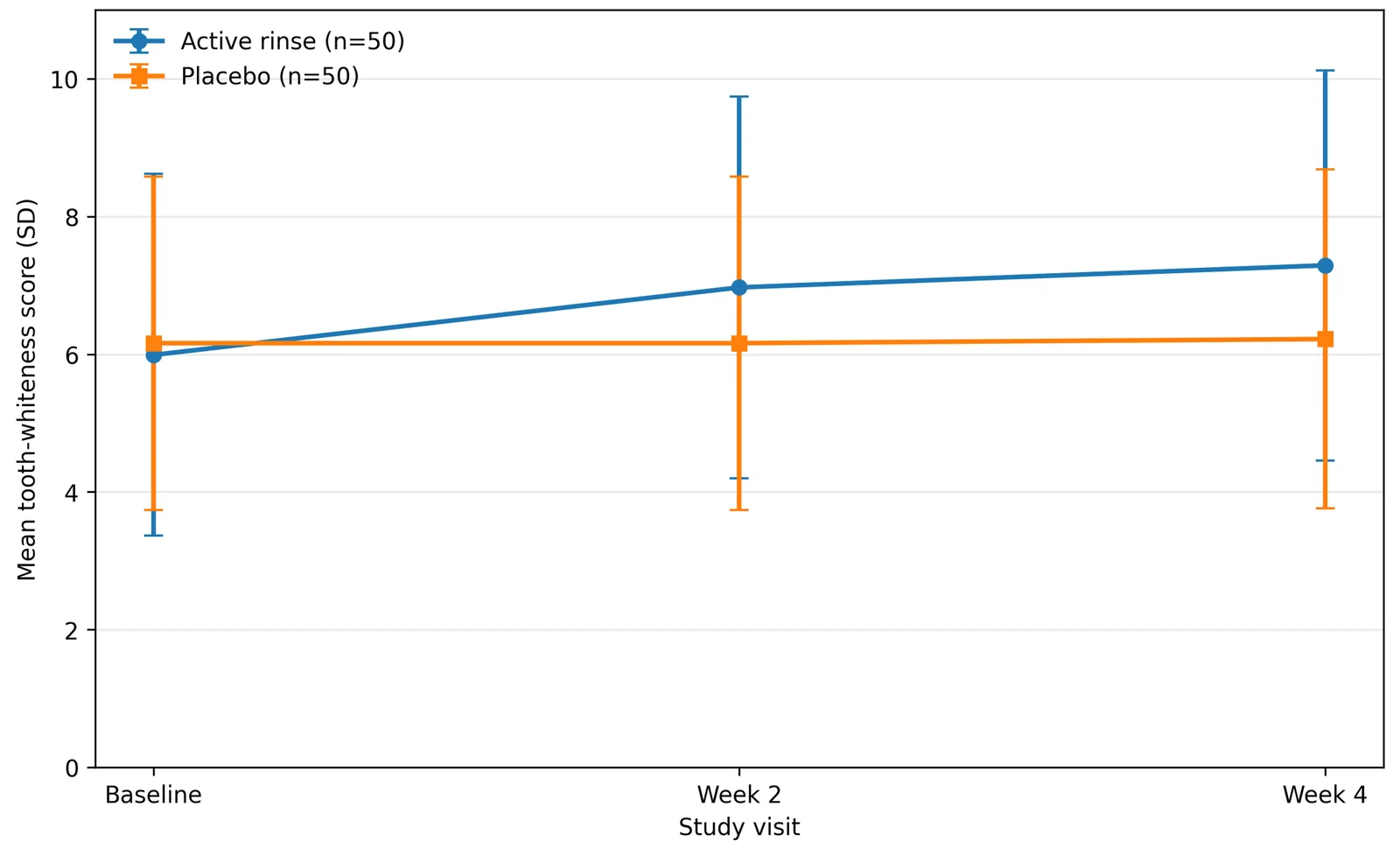
Mean tooth-whiteness scores at baseline, Week 2, and Week 4 in the active-rinse and placebo groups

Compliance, tolerability, and satisfaction

Treatment compliance was excellent and identical in both arms, with mean (SD) compliance of 100.00% (0.000) in each group; all 50 participants in each group achieved 100% compliance, and no participant fell below the 80% adherence threshold specified for withdrawal. Overall tolerability was rated Excellent by 46 (92.0%) active-group and 45 (90.0%) placebo-group participants, with the remainder rating tolerability as Good (4 (8.0%) and 5 (10.0%), respectively); no participant in either group rated tolerability as Fair or Poor.

The six-item overall treatment satisfaction questionnaire revealed a marked between-group divergence (Table 4). In the active group, 48 (96.0%) participants reported being Very Happy and 2 (4.0%) Happy with their treatment overall, and 50 (100.0%) reported being Very Happy or Happy across all six domains assessed, including convenience, perceived improvement, comparison with prior treatments, willingness to recommend, and willingness to continue treatment. In contrast, placebo-group participants predominantly reported being Not Happy, ranging from 43 (86.0%) to 50 (100.0%) participants across the six items, most commonly attributing their dissatisfaction to the absence of visible improvement in tooth whiteness.

**Table 4:** Overall treatment satisfaction questionnaire responses at end of study

Questionnaire item	Active, Very happy/Happy n (%)	Placebo, Not happy n (%)
Satisfaction with current treatment	50 (100.0)	43 (86.0)
Convenience of treatment	50 (100.0)	43 (86.0)
Improvement of dental condition	50 (100.0)	50 (100.0)
Rating vs previous treatments	50 (100.0)	50 (100.0)
Would recommend to others	50 (100.0)	50 (100.0)
Willingness to continue treatment	50 (100.0)	50 (100.0)

Safety

No adverse events, serious or otherwise, were reported in either treatment group throughout the four-week study. Physical examination findings (general appearance; head, eyes, ears, nose, and throat; abdominal; dermatological; respiratory; cardiovascular; gastrointestinal; musculoskeletal; and neurological systems) were normal in 50 (100%) participants in each group at every visit, with no abnormal findings recorded at screening, Week 2, or Week 4. Oral mucosal examination of the teeth, gums, tongue, and palate was similarly normal in all participants at all visits in both groups, with no evidence of mucosal irritation, gingival inflammation, or soft-tissue reaction attributable to the active rinse or its essential-oil constituents.

Haematology (haemoglobin, total and differential white cell counts, red cell count, packed cell volume, and platelet count) and biochemistry (SGOT, SGPT, and serum creatinine) parameters remained within normal limits from screening to Week 4 in both groups, with no clinically significant abnormalities identified in any participant. Vital signs (temperature, heart rate, respiratory rate, and systolic and diastolic blood pressure) likewise showed no clinically meaningful change over the study period in either arm. Urine pregnancy testing was negative in all tested female participants at both screening and Week 4 (Table 5).

**Table 5:** Summary of safety outcomes

Safety parameter	Active (n=50)	Placebo (n=50)
Treatment-emergent adverse events, n	0	0
Physical examination abnormalities (all visits), n	0	0
Oral mucosal examination abnormalities (all visits), n	0	0
Clinically significant laboratory values, n	0	0
Clinically significant vital sign changes, n	0	0
Drug compliance, mean % (SD)	100.00 (0.000)	100.00 (0.000)
Overall tolerability rated Excellent, n (%)	46 (92.0)	45 (90.0)
Overall tolerability rated Good, n (%)	4 (8.0)	5 (10.0)
Subjects completing study, n (%)	50 (100.0)	50 (100.0)

## Discussion

In this double-blind, randomised, placebo-controlled trial, the active oil pulling oral rinse containing MCT oil and a blend of essential oils produced a statistically significant improvement in tooth whiteness relative to placebo over 4 weeks of once-daily use. The magnitude of improvement increased progressively across the treatment period--from screening to Week 2, and further from Week 2 to Week 4--suggesting continued improvement with increasing duration of use. This pattern is consistent with the proposed role of mechanical oil swishing in oral hygiene and surface-stain removal [17]. The antimicrobial activity of essential-oil constituents provides an additional biologically plausible mechanism that could contribute to changes in the oral environment [19]. These mechanisms differ from the oxidative chemical bleaching produced by peroxide-based agents, which involves peroxide penetration and oxidation of chromogenic compounds within dental tissues [6-8].

Importantly, this efficacy was achieved without an observed safety signal during the four-week study. No adverse events were reported in either group, and no clinically significant changes in physical or oral examination findings, vital signs, or laboratory parameters were observed in either group. The absence of observed tooth sensitivity or gingival irritation in the present study is notable because adverse effects have been reported with conventional over-the-counter whitening products [9,10]. Reviews of peroxide-based whitening have also described treatment-related soft-tissue effects and other tolerability concerns [11,15]. Experimental and biological evidence has further raised concerns regarding the effects of peroxide-containing whitening materials on dental tissues, including potential enamel alterations [11,16]. The favourable tolerability observed in the present four-week trial therefore supports further evaluation of non-peroxide approaches, although the relatively short follow-up limits conclusions regarding longer-term safety. The high rates of excellent tolerability (92% in the active group and 90% in the placebo group) and 100% treatment compliance in both arms further support the acceptability and ease of use of the tested rinse within the conditions of this study.

The between-group divergence in the satisfaction questionnaire is noteworthy: while tolerability was similarly rated as excellent or good in both arms (indicating that both formulations were well tolerated), satisfaction with treatment outcome was overwhelmingly positive only in the active group, tracking closely with the objective shade improvement and providing convergent, participant-reported support for the primary efficacy finding.

These findings should be interpreted in light of several methodological and clinical limitations. The trial was conducted at a single centre over four weeks; therefore, the persistence of the whitening effect after treatment and its generalisability to broader populations remain to be evaluated. Tooth shade was assessed visually using the VITA classical shade guide under standardised conditions [21]. However, the VITA classical shade guide was not specifically designed to assess bleaching-related changes, and the use of the VITA Bleachedguide 3D-MASTER or instrumental colour measurements may have improved sensitivity to subtle changes in tooth whiteness. Visual shade matching is inherently subjective. Although an additional retrospective analysis demonstrated high temporal consistency in repeated shade assessments, this analysis does not establish formal examiner calibration or inter-examiner reliability. Furthermore, natural daylight may vary across assessment visits despite efforts to standardise the assessment environment. These factors may have introduced measurement variability and limited the reproducibility of the shade assessments. Although the active group demonstrated a statistically significant improvement in tooth-whiteness scores compared with placebo, statistical significance does not necessarily establish clinical significance. The mean score in the active group increased from 5.99 at baseline to 7.29 at Week 4, representing a 1.30-point increase on the 16-point scale. The clinical perceptibility and importance of this change were not independently established; therefore, the findings should be interpreted as evidence of a measurable improvement in visually assessed tooth shade rather than proof of substantial bleaching comparable to conventional peroxide-based treatments.

The eligibility criteria excluded participants with recent scaling, polishing, or whitening procedures and individuals who could not avoid specified stain-causing substances during the study period, but standardised professional prophylaxis before baseline assessment was not established. Differences in residual extrinsic surface staining or dietary exposure may therefore have contributed to variation in tooth-shade scores. Future studies should consider standardising baseline assessment procedures and documenting relevant dietary and behavioural exposures.

Finally, the substantial difference in patient-reported satisfaction between the treatment groups should be interpreted cautiously. Although the study was double-blind, differences in taste, aroma, or other sensory characteristics between the active essential-oil-containing rinse and the placebo formulation may have allowed some participants to infer their treatment allocation. Possible sensory unblinding may therefore have influenced subjective satisfaction ratings, including perceived convenience and willingness to recommend or continue treatment. Accordingly, satisfaction findings should be considered supportive patient-reported outcomes rather than independent confirmation of whitening efficacy.

Although the study was adequately powered for the prespecified efficacy endpoint, larger, multi-centre studies with longer follow-up would provide additional evidence regarding safety, durability, and generalisability. Future trials should also incorporate bleaching-specific shade guides or objective instrumental colour measurements, documented examiner calibration and reliability assessment, controlled lighting conditions, and systematic evaluation of blinding and potential staining-related confounders to better establish the magnitude and clinical relevance of the observed whitening effect.

Within these limitations, the findings contribute to the literature on non-peroxide approaches to tooth whitening. Previous research has examined oil pulling as an oral-health practice and has proposed potential effects related to oral hygiene and microbial control [17]. Experimental evidence also indicates that essential oils can demonstrate antimicrobial activity against oral pathogens [19]. The present study extends this literature by evaluating a standardised MCT-based oil pulling formulation containing a defined blend of essential oils in a randomised, placebo-controlled clinical setting and by demonstrating a measurable improvement in tooth-whiteness scores over four weeks.

Given the documented tolerability concerns associated with conventional bleaching agents, this type of oil pulling rinse may provide a useful option for individuals seeking gradual, no-peroxide cosmetic whitening, particularly those with sensitivity concerns or preference for natural-origin oral care products. Further studies across broader populations and longer follow-up periods would help confirm and extend these findings.

## Conclusions

In this randomised, double-blind, placebo-controlled trial, four weeks of once-daily use of the tested coco-mint oil pulling oral rinse containing MCT oil and an essential-oil blend produced statistically significant, progressive improvement in tooth whiteness compared with placebo. The active rinse demonstrated excellent compliance, tolerability, and high participant satisfaction, and no reported adverse events or clinically significant oral findings. These findings indicate that the tested formulation improved visually assessed tooth-whiteness scores over four weeks compared with placebo. However, the clinical significance and durability of the observed improvement require confirmation in larger, multi-centre studies using standardised shade assessment and objective colour measurements.

## References

[1] Christensen GJ (2005). Are snow-white teeth really so desirable?. J Am Dent Assoc.

[2] Carey CM (2014). Tooth whitening: what we now know. J Evid Based Dent Pract.

[3] Joiner A, Luo W (2017). Tooth colour and whiteness: a review. J Dent.

[4] Fasanaro TS (1992). Bleaching teeth: history, chemicals, and methods used for common tooth discolorations. J Esthet Dent.

[5] Haywood VB, Heymann HO (1989). Nightguard vital bleaching. Quintessence Int.

[6] Cooper JS, Bokmeyer TJ, Bowles WH (1992). Penetration of the pulp chamber by carbamide peroxide bleaching agents. J Endod.

[7] McCaslin AJ, Haywood VB, Potter BJ, Dickinson GL, Russell CM (1999). Assessing dentin color changes from nightguard vital bleaching. J Am Dent Assoc.

[8] Naidu AS, Bennani V, Brunton JM, Brunton P (2020). Over-the-counter tooth whitening agents: a review of literature. Braz Dent J.

[9] De Freitas M, De Carvalho M, Liporoni P (2021). Effectiveness and adverse effects of over-the-counter whitening products on dental tissues. Front Dent Med.

[10] Demarco FF, Meireles SS, Masotti AS (2009). Over-the-counter whitening agents: a concise review. Braz Oral Res.

[11] Omar F, Ab-Ghani Z, Rahman NA, Halim MS (2019). Nonprescription bleaching versus home bleaching with professional prescriptions: which one is safer? A comprehensive review of color changes and their side effects on human enamel. Eur J Dent.

[12] Grillon M, Di Bella E, Krejci I, Ardu S (2023). In vitro evaluation of tooth-whitening potential of peroxide-free OTC dental bleaching agents. Dent J (Basel).

[13] Permata D (2024). Bright smiles, hidden dangers: a literature review on the potential side effects of peroxide-based over-the-counter teeth whitening treatments. Int J Res Rev.

[14] Alkahtani R, Stone S, German M, Waterhouse P (2020). A review on dental whitening. J Dent.

[15] Li Y, Greenwall L (2013). Safety issues of tooth whitening using peroxide-based materials. Br Dent J.

[16] Li Y (1996). Biological properties of peroxide-containing tooth whiteners. Food Chem Toxicol.

[17] Naseem M, Khiyani MF, Nauman H, Zafar MS, Shah AH, Khalil HS (2017). Oil pulling and importance of traditional medicine in oral health maintenance. Int J Health Sci (Qassim).

[18] Schemehorn BR, Moore MH, Putt MS (2011). Abrasion, polishing, and stain removal characteristics of various commercial dentifrices in vitro. J Clin Dent.

[19] Hans VM, Grover HS, Deswal H, Agarwal P (2016). Antimicrobial efficacy of various essential oils at varying concentrations against periopathogen Porphyromonas gingivalis. J Clin Diagn Res.

[20] Schulz KF, Altman DG, Moher D (2010). CONSORT 2010 statement: updated guidelines for reporting parallel group randomised trials. BMJ.

[21] O'Brien WJ, Groh CL, Boenke KM (1989). One-dimensional colour order system for dental shade guides. Dent Mater.

